# High frequency microcloning of *Aloe vera* and their true-to-type conformity by molecular cytogenetic assessment of two years old field growing regenerated plants

**DOI:** 10.1186/1999-3110-54-46

**Published:** 2013-10-18

**Authors:** Sk Moquammel Haque, Biswajit Ghosh

**Affiliations:** Plant Biotechnology Laboratory, Department of Botany, Ramakrishna Mission Vivekananda Centenary College, Rahara, Kolkata, 700118 India

**Keywords:** *Aloe vera* leaf gel, Diploid and haploid karyotype, Meiotic study, Micropropagation, RAPD fingerprinting, True-to-type regenerants

## Abstract

**Background:**

*Aloe vera* (L.) Burm.f is an important industrial crop, which has enormous application in pharmaceutical, cosmetic and food industries. Thereby, the demand for quality planting material of *A. vera* is increasing worldwide. Micropropagation is the widely accepted practical application of plant biotechnology that has gained the status of a multibillion-dollar industry throughout the world and this techniques can be used to meet the industrial demand of *A. vera*. Present studies aim to develop a proficient methods of high-frequency true-to-type plantlet regeneration without intermediate callus phase for *A. vera.*

**Results:**

Nodal portion of rhizomatous stem of *A. vera* were cultured on Murashige and Skoog (MS) medium (Physiol. Plant. 15:473 – 497, 1962) supplemented with various cytokinin and *A. vera* leaf gel (AvG) as organic supplement. Number of proliferated shoots per explant was increased along with the regeneration cycles and on MS medium supplemented with 2.5 mg/L 6-benzylaminopurine and 10.0% (v/v) AvG, only 17.8 ± 0.35 shoots per explant were induced on 1^st^ regeneration cycle whereas on 3^rd^ regeneration cycle these number increase to 38.5 ± 0.44 shoots per explant on the same medium composition. AvG have an encouraging role to increase the proliferation rate and on 3^rd^ regeneration cycle 27.6 ± 0.53 shoot per explant induced on 2.5 mg/L BAP, but these number increase to 38.5 ± 0.44 shoots per explant when 10.0% (v/v) AvG was added along with 2.5 mg/L BAP. After transfer of individual excised shoots to a one-third strength MS medium containing 20.0% (v/v) AvG, all the shoots formed whole plantlets with maximum number (9.6 ± 0.29) of roots per shoot. 95.0% of the regenerated plantlets survived on poly-green house. Normal flower appeared in 84.2% field growing micropropagated plants after 18 to 20 months of field transfer. Further, clonal fidelity of the two years old micropropagated plants was established by studying mitotic and meiotic chromosomal behavior and also considered the chromosome number and structural organization. There were no alterations in chromosome phenotypes, somatic haploid (pollen mitosis) and diploid chromosome count (n = 7; 2n = 14), or meiotic behavior. Randomly amplified polymorphic DNA analyses revealed there were no somaclonal variations among these regenerants.

**Conclusions:**

These results confirm the very reliable method for large scale production of true-to-type plantlets of *A. vera,* which can be used for commercial purpose.

**Electronic supplementary material:**

The online version of this article (doi:10.1186/1999-3110-54-46) contains supplementary material, which is available to authorized users.

## Background

*Aloe* is an important commercial crop available in a wide range of species and varieties in international markets. *A. vera* has been used for medicinal purposes in several cultures of different countries: India, China, Japan, Greece, Egypt and Mexico for millennia (Marshall 1990). Different properties being attributed to the inner, colorless, leaf gel and to the exudate from the outer layers of *Aloe* leaf in a number of studies for several years (Reynolds and Dweck 1999 Ni et al. 2004; Liu et al. 2011). Due to the huge utilization in pharmaceutical, cosmetic and food industries (Vogler and Ernst 1999; Eshun and He 2004; Botes et al. 2008; Grace et al. 2008; Bedini et al. 2009; Rodríguez et al. 2010; Chen et al. 2012; Lad and Murthy 2013; Zapata et al. 2013), the demand for quality planting material of *A. vera* is increasing day-to-day. Mass propagation of uniform, healthy plants through tissue culture is the only viable technique for production of large numbers of clonal plants in a short time. Several attempt was taken for last few decades to develop tissue culture systems of *Aloe* spp. (Meyer and van Staden 1991; de Oliveira and Crocomo 2009; Singh et al. 2009; Das et al. 2010a; Gantait et al. 2011; Rathore et al. 2011b; Amoo et al. 2012 2013), but still the efficient regeneration protocols are requisite to large scale production of true-to-type plants of this commercially important species. Aim of our present studies is to develop a proficient and cost effective method for rapid and high frequency shoot multiplication and in vitro rooting of *A. vera* from rhizomatous stem explants. The genetic fidelity of micropropagation system needs to be ascertained before using it at commercial level (Goswami et al. 2013). Prior to the availability of DNA-based markers; cytological, morphological and agronomic traits were exploited for the selection of the superior genotypes. However, morphological markers are not considered reliable because they are affected by environmental and cultivation conditions. In latest studies, cytogenetic observation of micropropagated plants was investigated for the conformity of chromosomal change in structural or ploidy level (Das et al. 2010b; Rana et al. 2012; Das et al. 2013). Molecular markers are more powerful tools for studying genetic diversity and relationships between genotypes. RAPD fingerprinting can be used to trace genetic or epigenetic changes at the genome level (Arnholdt-Schmitt and Schaffer 2001; Leelambika and Sathyanarayana 2011). In recent years, RAPD based detection of genetic polymorphism have been found successful application in describing somaclonal variability/homogeneity of micropropagated individual of many plant species (Savita et al. 2012; Paridaa et al. 2013; Goswami et al. 2013; Cheruvathur et al. 2013; Kumar et al. 2013; Haque and Ghosh 2013a). Manipulation of the composition and ratio of plant growth regulators (PGRs) is often the primary empirical approach used for optimization of in vitro micropropagation methods (Shukla et al. 2012). The present study was thus aimed at the following: (1) induction and regeneration of plants via direct shoot regeneration, (2) RAPD profiles analysis and (3) comparative cytogenetic assessment of two years old micropropagated plants and mother plant.

## Methods

### Shoot regenerations

*Aloe vera* (L.) Burm.f. plants growing in wild conditions were collected during September 2010 from Nallamalas ranges of the Eastern Ghats Mountains of the Andhra Pradesh state of India and maintained in our experimental garden. After removing all leaves, the rhizomatous stem were used as explant and washed with 2.0% (w/v) systematic fungicide (Thiram) for 25 min followed by 2.5% liquid detergent (Tween-20 solution) for 3 min and then surface-sterilized with freshly prepared 0.15% (w/v) aqueous solution of mercuric chloride (HgCl_2_) for 12 min and rinsed 3 times with sterile distilled water to remove traces of HgCl_2_. The explants (≈8 mm piece of rhizomatous stem from nodal portion containing axillary shoot bud) were cultured on MS (Murashige and Skoog 1962) basal medium containing 3.0% (w/v) sucrose and various concentration and combination of cytokinin [6-benzylaminopurine (BAP), Kinetin (KIN)] and *Aloe vera* leaf gel (AvG). For AvG preparations, mature fresh leaves of *A. vera* ware collected from experimental garden and kept half an hour to remove yellow liquid exudate, then washed thoroughly in running water. Then leaf skin was removed and the odorless, colorless mucilaginous leaf gel was peeled off with the help of stainless steel spoon and were homogenized in mixture-grinder. Then the homogenates were filtered with tea-net and this liquid was termed as ‘AvG’, which was stored at 4°C until use. AvG contains over 75 active ingredients (Hamman 2008) and serve as a nutritional supplement. The cultures were incubated in growth chamber maintained at 23 ± 2°C under a 16 h photoperiod with a photosynthetic photon flux density of approximately 50 μmol m^-2^ s^-1^ emitted from cool fluorescent tubes (Philips India Ltd.). At every 4 weeks intervals, the cultures were sub-cultured in their respective fresh media. After completion of every regeneration cycle (8 weeks), each individual shoots (≥ 2.0 cm) were separated from proliferated shoot clumps for in vitro rooting and then pre-existing explants were re-inoculated in their respective fresh media for next regeneration cycle.

### Root induction of microshoots

Regenerated shoots (2.0-4.0 cm long) with 3-4 leaves were separated from clumps into single ones and were cultured on only agar-water medium (without any MS nutrients and sucrose) and three different strength of MS medium (full strength, two-third strength and one-third strength) supplemented with 3.0%, 2.0% and 1.0% sucrose respectively. Similarly, the effect of different concentrations of AvG (0%-40.0%) was also evaluated on rooting efficiency of microshoots.

### Acclimatizing and field evaluation of regenerated plants

Rooted plantlets (about 6-8 cm) were transferred to small earthen pots containing ‘Soilrite’ (sterile, chemically inert horticultural graded perlite marketed by Keltech Energies Ltd., Bangalore, India) and covered with transparent polythene bags to maintain 90-99% relative humidity and were kept in 25 ± 2°C temperature and 16-h photoperiod for 25 to 30 days. Thereafter, the acclimatized plants were transplanted on earthen tubs containing a mixture of soil and vermin compost (3:1 ratio) and maintained inside the poly-green house (30 ± 2°C temperature and relative humidity of 60-65%) for another 3 months. Finally the plants were transferred to the field under full sunlight.

### Mitotic karyotype study

In vivo mother plant and field grown two years old ex vitro micropropagated plants were used for cytological analysis. Total 25 root tips of mother plant as well as 125 root tips of 25 randomly selected micropropagated plants were excised, washed with tap water, and pre-treated with a saturated solution of Þ-dichlorobenzene for 4 h at 16-18°C. Pre-treated material was thoroughly washed with tap water, fixed in an ethanol/acetic acid solution (3:1; v/v) for 24 h at 4°C. For somatic chromosome counts and karyotypic analysis, fixed root tips were stained with 2.0% aceto-orcein: 1 (N) HCl (9:1 v/v) mixture followed by incubating for 2 h at room temperature. Then stained root tips were macerated and squashed in 45.0% acetic acid. Chromosome plates were observed in Leica DM750 microscope and photographed with Leica DFC295 camera. Minimum of 5 metaphase plates from each root tip were analyzed to determine the somatic chromosome number at the metaphase stage.

### Meiosis & pollen mitosis study

For meiotic and pollen mitotic studies, young inflorescences were fixed at the appropriate stage in a fixative containing ethanol/acetic acid (3:1; v/v) for 24 h at 12-15°C. Smear preparations were made in 2.0% aceto-carmine following Sharma and Sharma’s (1980) methods. All the meiotic and pollen mitotic plates were observed in Leica DM750 microscope and photographed with Leica DFC295 camera.

### Genomic DNA extraction

Genomic DNA was extracted from leaf tissue (excluding transparent gel like region) of both mother plant and 10 randomly selected field grown two years old micropropagated plants separately using CTAB protocol (Doyle and Doyle 1990) with slight modification. Fresh leaf tissue (≈100 mg) was grinded to powder in liquid nitrogen using mortar and pestle. Powdered tissue was placed in 1.0 ml of pre-warmed (65°C) extraction buffer (2.5% w/v CTAB, 1.5 M NaCl, 25 mM EDTA, 100 mM Tris HCl pH 8.0, 1.0% w/v polyvinylpyrrolidone) in a 1.5 ml microcentrifuge tube. Just prior to homogenization, 2.0 μl of β-mercaptoethanol was added to the tube and these were incubated at 65°C for 60 min. Immediately following homogenization centrifuged (1000 × g at 22°C) for 10 min and the supernatant was transferred to fresh 2.0 ml microcentrifuge tube. Then equal volume of chloroform: isoamyl alcohol (24:1 v/v) was added and mixture was gently mixed for 10 min by inverting the tube. Then centrifuged (1000 × g at 22°C) for 8 min to separate phases. The upper aqueous phase was transferred to a fresh microcentrifuge tube and repeats the chloroform isoamyl alcohol (24:1 v/v) step. DNA was precipitated with double volume of chilled ethanol for overnight at−20°C, then centrifuged (4,000 × g at 22°C) for 10 min. The pellet was air dried and re-suspended in 100.0 μl of Tris EDTA buffer. Then samples were treated with RNase at a final concentration of 50.0 ng/ml and incubated at 45°C for 60 min. Quality and quantity of DNA was monitored by spectrophotometry and gel inspection. Each sample was diluted at concentrations ranging from 45.0-55.0 ng/μl and stored at−20°C.

### RAPD analysis

PCR were carried out in a total volume of 20.0 μl containing 50.0 ng of genomic DNA, 200 μM of the dNTP mix (Sigma), 1 X Taq buffer-A and 1 unit Taq DNA polymerase (GeNei™). All constituents except primer and DNA were prepared as 1 X master mix. Amplification was carried out in DNA Thermal Cycler (MJ Mini™, Bio-Rad). PCR used an initial denaturation of 94°C for 5 min, followed by 40 cycles of 94°C for 45 sec, 38-43°C for 60 sec and 72°C for 90 sec. A final extension step of 7 min at 72°C was included after the last cycle. A total 32 primers from OPA, OPC, OPG, OPJ, OPK, OPL, OPM, OPN, OPAC, OPAD, OPAE, OPAF series (Operon Technologies Inc, Alameda, USA) were used for amplification using the cycling conditions mentioned above. The amplified products (20.0 μl) were mixed with 4.0 μl of 6 X DNA loading dye (GeNei™) and were electrophoresed along with ‘100 bp Plus’ DNA ladder (Thermo Scientific) in a horizontal gel apparatus (PowerPack™ Basic, Bio-Rad) using 2.5% agarose gel (containing ethidium bromide) in 1 X Tris-acetate-EDTA buffer pH 8.0 at 60 Volt for 120 min. The gels were visualized and photographed using a Gel Documentation system (Gel Doc™ XR, Bio-Rad). All PCRs were repeated thrice to check their reproducibility. Only consistently reproducible, well resolved fragments were scored.

### Statistical analysis

Each treatment contained three replicates with 10 explants per replicate. The data pertaining to the number of shoots or roots per explant were subjected to a one-way analysis of variance (ANOVA). The differences among the means were compared by high-range statistical domain using Duncan’s test with the standard software SPSS 16.0 version.

## Results and discussion

### Effect of PGRs and AvG on shoot regeneration

**Figure 1:**
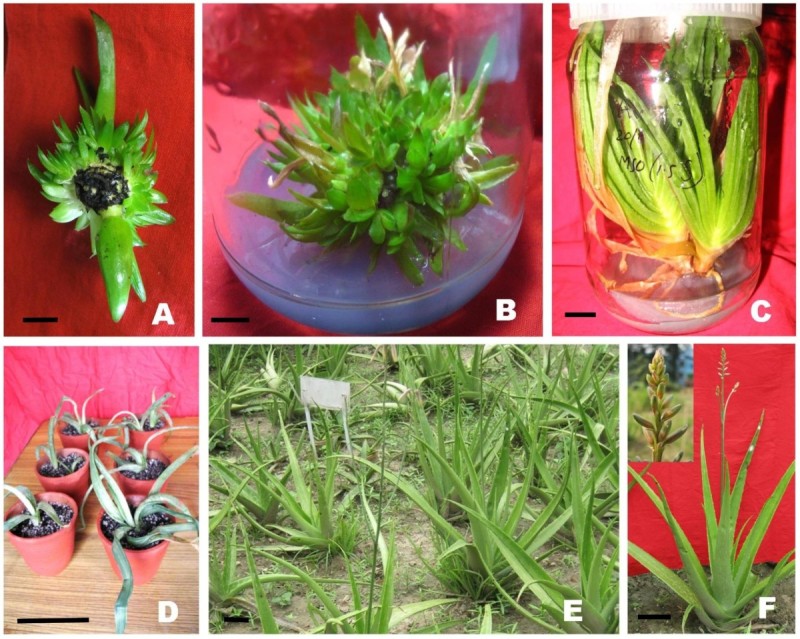
**Different stages of Micropropagation and field performance of** ***Aloe vera*** **. (A)** Multiple shoots induced in MS medium supplemented with 2.5 mg/L BAP on third regeneration cycle (bar = 1 cm). (**B**) Multiple shoots induced in MS medium supplemented with 2.5 mg/L BAP and 10.0% AvG on third regeneration cycle (bar = 1 cm). (**C**) Complete plantlets with root system (bar = 1 cm). (**D**) Hardening of regenerated plants (bar = 10 cm). (**E**) Field grown regenerated plants of 18 months old (bar = 10 cm). (**F**) 22 months old regenerated plant with inflorescence (bar = 10 cm).

The effects of different cytokinin types and concentrations on the explant for shoot induction was evaluated up to three regeneration cycle and shown in Table 1. High frequency of shoot regeneration from explant (ranging from 73.3% to 100%) was obtained in all the treatments, excluding the control where only one shoot induced. Initiation of shoot buds were observed in naked eye within 26-35 d of implantation depending on the types and concentrations of PGRs. On 1^st^ regeneration cycle, maximum 14.5 ± 0.31 and 9.7 ± 0.29 number of shoots were induced on MS medium containing 2.5 mg/L BAP and 4.0 mg/L KIN respectively after 8 weeks of culture. These number of regenerated shoots per explant was increased along with each regeneration cycles and on 3^rd^ regeneration cycle, maximum 27.6 ± 0.53 number of shoots per explant are produced on medium containing 2.5 mg/L BAP after 8 weeks of culture (Figure 1A), whereas maximum 20.3 ± 0.33 shoots produced on 4.0 mg/L KIN containing medium after same duration. Hence, BAP proved to be more effective for multiplication of shoots as compared to KIN. More effective response of BAP over other cytokinins on propagation of *Aloe* sp. was earlier reported by Singh et al. (2009). Beyond the optimum concentration, there was a decrease in shoot production with an increased BAP concentration (Table 1). There was sharp increase in the number of shoots produced per explant as well as shoots with length greater than 2.0 cm when AvG was supplemented along with optimal concentration of BAP. Many complex organic supplements like coconut water, banana powder, tomato and orange juice have beneficial effects on in vitro plant cell and tissue cultures and widely used for enhancement of in vitro growth of many plants (Molnár et al. 2011). In the present study AvG is used as another complex organic supplements for the enhancement of proliferation rate. At each concentration of AvG treatments, the number of shoot induction was higher in compared to that of only BAP. MS medium supplemented with 2.5 mg/L BAP and 10.0% AvG triggered the maximum number (17.8 ± 0.35) of shoots (length ≥ 2 cm) in 1^st^ regeneration cycle. These numbers was increased up to 38.5 ± 0.44 after 3^rd^ regeneration cycle on the same medium composition (Figure 1B). It is noted that AvG was used as a supplementary source of organic and inorganic ingredients for better growth and production of healthy in vitro plants of *Bacopa* (Haque and Ghosh 2013b). *Aloe* gel contains 5.43% (w/w) total sugar, 36.0% of which was quantified as glucose, 18.0% as fructose and the remainder as maltose and sucrose (Ni et al. 2004; Botes et al. 2008). In addition to the different carbohydrates, *AvG* contains 75 potentially active constituents including vitamins, enzymes, minerals, lignin, saponins, salicylic acids, amino acids and different inorganic salts (Vogler and Ernst 1999; Hamman 2008), which may enhance the multiplication rate of *A. vera*.

**Table 1:** **Effect of cytokinins and** ***Aloe vera*** **leaf gel (AvG) supplemented with MS basal medium on shoots regeneration of** ***Aloe vera***

MS medium + Supplement		1^st^regeneration cycle		3^rd^regeneration cycle	
Supplement types	Supplement Conc.	Response (%)	Number of shoot (≥ 2.0 cm) per explant	Response (%)	Number of shoot (≥ 2.0 cm) per explant
***Control***					
Without PGRs/AvG	0	0	0.0 ± 0.0^a^	93.3	1.0 ± 0.0^a^
***Cytokinin (mg/L)***					
BAP	1.0	73.3	8.3 ± 0.23^c^	100	16.2 ± 0.34^c^
	2.5	80.0	14.5 ± 0.31^f^	100	27.6 ± 0.53^h^
	4.0	66.7	11.7 ± 0.44^e^	100	23.1 ± 0.44^f^
KIN	1.0	63.3	5.2 ± 0.24^b^	100	12.8 ± 0.28^b^
	2.5	76.7	8.2 ± 0.24^c^	100	17.7 ± 0.40^d^
	4.0	70.0	9.7 ± 0.29^d^	100	20.3 ± 0.33^e^
***Cytokinin (mg/L) + AvG (%)***					
BAP + AvG	2.5 + 5	93.3	15.9 ± 0.24^g^	100	33.3 ± 0.41^j^
	2.5 + 10	96.7	17.8 ± 0.35^h^	100	38.5 ± 0.44^k^
	2.5 + 15	93.3	14.3 ± 0.28^f^	100	30.9 ± 0.43^i^
	2.5 + 20	90.0	12.5 ± 0.30^e^	100	24.3 ± 0.41^g^

Each value represents the means ± SE, *n* = 30. Means followed by the same letters in each column are not significantly different at *P* < 0.05 according to Duncan’s multiple range tests.

During the initiation of culture the rhizomatous stem explants of *A. vera* exhibited excessive leaching of phenolic substances, a cause of browning of the culture medium when cultured on only cytokinin containing medium. But this problem was overcome when AvG supplemented along with BAP. According to earlier findings of Singh et al. (2009), incorporation of antioxidants (viz. citric acid, ascorbic acid, polyvinylpyrrolidone) to the culture medium promoted growth and prevented browning of the culture medium for *A vera* micropropagation. We know, along with the nutritional supplementary activity, AvG have strong antioxidant properties (Botes et al. 2008; Amoo et al. 2012,2013). Thereby, addition of AvG may serve antioxidants activities in the culture medium which not only minimized the browning of tissues but also reduced leaching of phenolic compounds, which is harmful for in vitro cultures.

### Effect of AvG and nutritional strength of medium on root induction

All three strength of MS medium (1, 2/3, 1/3) with 3.0%, 2.0%, and 1.0% sucrose respectively or even only agar-water medium resulted in root induction with frequencies ranging from 20.0% to 76.7% (Figure 1C). A higher number of roots per cultured shoot were obtained with AvG (10-40%) treatment when compared with 1, 2/3, 1/3 strength of MS medium with 3.0%, 2.0%, and 1.0% sucrose respectively or nutrient free agar-water. The maximum number of roots (9.8 ± 0.29) with cent percent response frequency and the longest root (3.1 ± 0.10) were recorded within 18 d of implantation on 1/3 strength MS medium supplemented with 20% AvG (Table 2). Though many workers previously described the use of auxin for in vitro rooting of *Aloe* sp*.* (Hashemabadi and Kaviani 2008; Amoo et al. 2012), but in present study no auxin supplements are used at any stage of this experiment, neither for plant regeneration nor for root induction purpose. The root inducing properties of AvG was previously reported on *Bacopa chamaedryoides* (Haque and Ghosh 2013b) and *Aloe vera* (Das et al. 2010a). According to findings of present study, addition of AvG to the medium not only increase the percentage of response and number of root per shoot but also the growth of the plantlets was improved which are corroborate with our previous studies on *Bacopa chamaedryoides* (Haque and Ghosh 2013b). So in this context the present study proposes an unique auxin free culture system for large scale propagation of *A. vera*, that substitute by addition of AvG–a less expensive PGR-like natural complex.

**Table 2:** **Effect of the strength of MS medium and concentration of sucrose (S) and** ***Aloe vera*** **leaf gel (AvG) on in vitro rooting of** ***Aloe vera*** **(after 18 d of implantation)**

Strength of MS medium, concentration of sucrose (w/v) and AvG (v/v)	Percentage of shoot showing root formation	Number of root per shoot [means ± SE]	Length of longest root per shoot (cm) [means ± SE]
Full MS + 3% S	56.7	3.3 ± 0.25^a^	1.5 ± 0.12^a^
Two third MS + 2% S	70.0	4.2 ± 0.23^b^	1.9 ± 0.08^b^
One third MS + 1% S	76.7	5.6 ± 0.26^c^	2.3 ± 0.08^c^
Water-agar medium*	20.0	2.7 ± 0.33^a^	2.9 ± 0.14^d^
One third MS + 1% S + 10% AvG	86.7	7.3 ± 0.24^d^	2.4 ± 0.07^c^
One third MS + 1% S + 20% AvG	100	9.8 ± 0.29^e^	3.1 ± 0.10^d^
One third MS + 1% S + 30% AvG	100	9.2 ± 0.26^e^	2.8 ± 0.08^d^
One third MS + 1% S + 40% AvG	93.3	6.5 ± 0.23^d^	2.1 ± 0.10^bc^

Each value represents the means ± SE, n = 30. Means followed by the same letters in each column are not significantly different at P < 0.05 according to Duncan’s multiple range tests.

*Medium without MS nutrient and sucrose but solidified with agar.

### Acclimatizing and field evaluation of regenerated plants

A total of 76 out of 80 (95.0%) in vitro rooted plantlets were successfully acclimatized for 25 to 30 days (Figure 1D). Thereafter, the acclimatized plants were transplanted on earthen tubs containing a mixture of soil and vermin compost (3:1 ratio) for next 3 months with 100% survival rate. Ultimately all plants were established in soil on field condition under full sunlight (Figure 1E). The majority of the micropropagation protocols do not deals with concern of the acclimatization process or they only mention that the acclimatization was tested with success, but we studied it thoroughly up to 2 years after acclimatizing. After 18 to 20 months of field transfer, 84.2% (64 out of 76) of the survived plants flowered normally (Figure 1F).

### Diploid and haploid karyotype analysis

**Figure 2:**
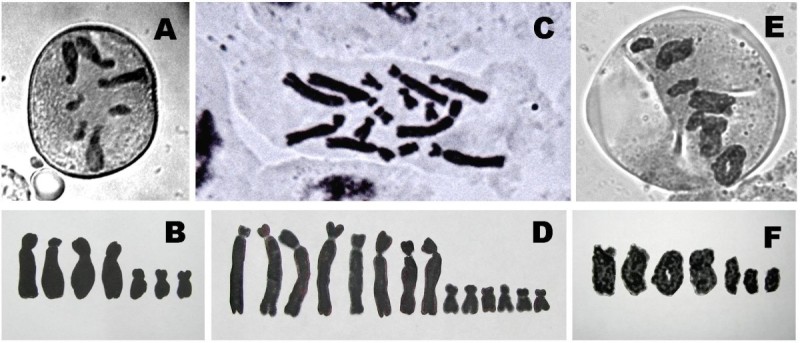
**Mitotic and Meiotic metaphase plates and karyogram of regenerated** ***Aloe vera*** **plants. (A)** Metaphase plate of pollen mitosis showing n = 7 chromosomes. **(B)** Karyogrm of haploid pollen grain. **(C)** Mitotic metaphase plate of root-tip cell showing 2n = 14 chromosomes. **(D)** Karyogram of diploid somatic cell. **(E)** Metaphase-I of meiosis of pollen mother cell showing 7 pairs of bivalent chromosomes **(F)** Karyogram of meiotic bivalent chromosomes.

The haploid chromosome number for *A. vera* was found to be n = 7 (Figure 2A). Three short chromosomes are sub metacentric ranging from 5.6 μm to 6.2 μm and four long chromosomes are acrocentric ranging from 12.8 μm to 16.7 μm. One long chromosome found with secondary constriction at their long arm. The haploid karyotypic formula is n = x = 7 (1st^sat^ + 3st + 3sm). The diploid chromosome number was found to be 2n = 14 (Figure 2C) with four pair of long acrocentric chromosomes ranging from 14.4 μm to 17.9 μm and three pair of short sub metacentric chromosomes ranging from 4.6 μm to 5.4 μm. Secondary constrictions are found at the long arm of one pair of long chromosome. The diploid karyotypic formula is 2n = 2x = 14 (2st^sat^ + 6st + 6sm). There were no anomalies in chromosome number or structure and organization for any of the regenerated plants. Das et al. (2010a) finds a tetraploid *A. vera* plants, which was induced spontaneously during in vitro culture, which fail to maintain cytogenetic stability and true-to-type of regenerants. But in our present study, all the regenerants are diploid, there was no evidence of any ploidy change. Karyogram of haploid and diploid mitotic chromosomes (Figure 2B, D) and bivalent meiotic chromosomes (Figure 2F) show distinct bimodal nature as source plant. All haploid and diploid karyotype data of in vivo grown mother plant and tissue culture raised plants of *A. vera* confirm they are karyotypically stable. Thorough characterization and classification of tissue culture induced chromosome aberrations have led to a better understanding of somaclonal variation (Lee and Phillips 1988; Bairu et al. 2011).

### Meiotic analysis

**Figure 3:**
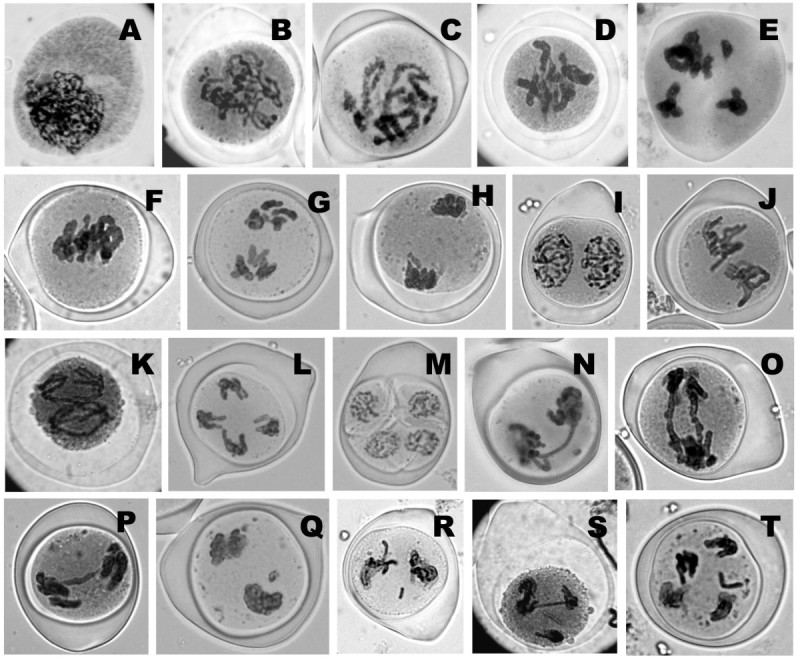
**Aceto-carmine stained normal (A-M) & abnormal (N-T) meiotic stages of** ***Aloe vera.*** **(A)** Leptotene stage. **(B)** Zygotene stage showing chromatin threads **(C)** Pachytene stage showing beaded chromosomes. **(D)** Diplotene stage showing ‘X’ shaped chiasma. **(E)** Diakinesis showing 7 bivalents with 4 long and 3 short chromosome pairs. **(F)** Side view of metaphase-I. **(G)** Side view of anaphase-I. **(H)** Side view of telophase-I. **(I)** Side view of prophase-II **(J)** Side view of metaphase-II. **(K)** Side view of anaphase-II. **(L)** Side view of telophase-II. **(M)** Tetrad showing 4 pollen grains. **(N)** Side view of anaphase-I with single chromosomal bridge and lagged chromosome. **(O)** Side view of anaphase-I with double chromosomal bridge. **(P)** Side view of anaphase-I with single chromosomal bridge **(Q)** Telophase-I with lagged chromosome. **(R)** Metaphase-II with lagged chromosome. **(S)** Telophase-II with single chromosomal bridge. **(T)** Telophase-II with univalent laggard chromosome.

The medicinal value and male sterility of *A. vera* make it important for cytological investigation. The behaviour of meiotic chromosomes was investigated in both mother plants and tissue culture raised plants of *A. vera*. All the various meiotic stages from leptotene to tetrad formation were studied (Figure 3A-M). The course of meiosis was normal in most of the cells but some meiotic irregularities have also been observed as mother plant. A beaded structure of bivalent chromosomes were observed on pachytene stage (Figure 3C). Seven bivalent with many chiasma are observed on diplotene stage (Figure 3D). Perfect chromosome pairing with 7 bivalents at diakinesis and metaphase-I (Figure 3E and Figure 2E) and with normal chromosomal segregation with 7:7 disjunctions at anaphase-I of meiosis (Figure 3G) was observed. At anaphase-I, one chromosome of each homologous migrates toward the opposite pole (Figure 3G). At anaphase-II, individual chromatids are separate and move toward four opposite poles (Figure 3K). However, in total 8.5% meiotic abnormalities in mother plants and 7.8% abnormalities in regenerated plants were observed in the form of ‘chromosome bridge’ (single and double bridge), ‘lagged chromosome’ on both meiosis-I and meiosis-II (Figure 3N-T). Our present findings are corroborate with the earlier report, where chromosomal deformities occurs spontaneously during meiosis of in vivo plants *A. vera.* (Vig 1968; Chaudhuri and Chaudhury 2012).

### RAPD analysis

**Figure 4:**
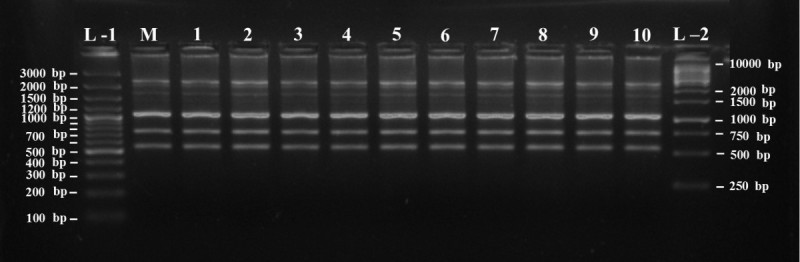
**RAPD banding profile of both mother plant and field grown micropropagated plants of** ***Aloe vera*** **using OPK-10 primer showing 6 monomorphic bands ranging from 600 bp to 2500 bp (Lane ‘L-1’ = 100 bp plus DNA ladder, Lane ‘M’ = Mother plant, Lane ‘1-10’ = ten different micropropagated plants, Lane ‘L-2’ = 1 kb DNA ladder).**

For the analysis of genetic stability, two years old ten micropropagated plants and a control parent plant were assessed through RAPD analysis. Total 32 primers were used for PCR amplification out of which 8 primers don’t give any amplification. Although 9 primers produce 1 or 2 prominent bands, but we consider only those primers which produce 3 or more bands. The 15 selected RAPD primers, sequence, total number of bands scored, and annealing temperature for each primer are varying between 38-41°C and summarized in Table 3. Total 82 bands are produced by all 15 selected primers with an average of 5.5 bands per primer. The number of bands for each selected primer varies from 3 to 10. The highest number of bands obtained was 10 in case of primers OPA-16 and OPM-06; and the lowest number of bands obtained was 3 in case of primers OPL-5 and OPAC-07. All bands generated by the RAPD techniques were monomorphic in nature, no polymorphic bands were observed (Figure 4). The size of monomorphic bands varies among different primers from ≈ 200 bp to ≈ 2500 bp. RAPD analysis revealed no evidence of genetic variation either within or between the micropropagated plants and the mother plant. Therefore, all the micropropagated plants were found to be genetically uniform and true-to-type with their parent. The somaclonal variations are common problem among micropropagated plants, which can be detected by various PCR-based techniques such as RAPD, simple sequence repeat (SSR), inter-simple sequence repeat (ISSR), and amplified fragment length polymorphism (AFLP) etc. However, in present studies the RAPD methods are used for rapid evaluation of somaclonal variability in tissue-cultured plants, by fast scanning of the whole genome. In recent report, genetic fidelity analysis of *A. vera* was carried out using RAPD fingerprinting (Samantaray and Maiti 2008; Rathore et al. 2011a). In present studies, along with the previously reported RAPD markers, we also detected some new RAPD marker for this plant species and both the cases only monomorphic bands are produced. Similar observation was reported in many other plants like *Tylophora, Desmodium, Catharanthus* where true-to-type nature of regenerated plants were confirmed by using RAPD markers (Haque and Ghosh 2013a; Cheruvathur et al. 2013; Kumar et al. 2013). Genome variation in tissue culture is of importance for commercial use in plant propagation as well as for basic research on plant growth and development (Arnholdt-Schmitt and Schaffer 2001). The results obtained suggested that direct organogenesis from rhizomatous stem explants of *A. vera* induced by BAP and enhanced by AvG carry no risk of generating somaclonal variants.

**Table 3:** **List of RAPD primers, their sequence, optimal annealing temperature (T**_**m**_ **) and banding pattern of both mother plant and field-grown micropropagated plants of** ***Aloe vera***

Sl. No.	Prime	Sequence 5′-3′	T_m_(°C)	Total Bands
1	OPA-09	GGGTAACGCC	41	5
2	OPA-16	AGCCAGCGAA	38	10
3	OPC-06	GAACGGACTC	41	6
4	OPG-08	TCACGTCCAC	38	4
5	OPG-10	AGGGCCGTCT	41	4
6	OPJ-04	CCGAACACGG	41	4
7	OPK-10	GTGCAACGTG	41	6
8	OPL-02	TGGGCGTCAA	41	9
9	OPL-04	GACTGCACAC	41	4
10	OPL-05	ACGCAGGCAC	41	3
11	OPM-06	CTGGGCAACT	41	10
12	OPN-15	CAGCGACTGT	38	6
13	OPN-18	GGTGAGGTCA	38	4
14	OPAC-07	GTGGCCGATG	38	3
15	OPAC-20	ACGGAAGTGG	38	4
**Total**				82

## Conclusions

In conclusion, according to present protocol, high frequency of plantlets production was achieved without use of any auxin on any stage throughout the study, i.e. from explant inoculation to plantlet hardening, a totally auxin free culture system. The molecular cytogenetic evidence of the genetic stability and true-to-type conformity of the regenerants of this protocol make it valuable for large-scale propagation of *Aloe vera* at industrial level. So in this contexts present findings are totally innovative and unique as compare to previous studies.

## Authors’ information

SMH is working as a Research Fellow under the guidance of BG. BG is an Associate Professor in the Plant Biotechnology Laboratory, Department of Botany, Ramakrishna Mission Vivekananda Centenary College, Kolkata, India.

## Authors’ original submitted files for images

Below are the links to the authors’ original submitted files for images. Authors’ original file for figure 1 Authors’ original file for figure 2 Authors’ original file for figure 3 Authors’ original file for figure 4

## Abbreviations

2n: Diploid number of chromosomes
AvG: *Aloe vera* leaf gel
BAP: 6-benzylaminopurine
CTAB: Cetyltrimethyl ammonium bromide
KIN: Kinetin
MS medium: Murashige and Skoog (Physiol. Plant. 15:473 – 497, 1962) basal medium
n: Haploid number of chromosomes
PCR: Polymerase chain reaction
PGRs: Plant growth regulators
RAPD: Randomly amplified polymorphic DNA.
