# Supplementary figures and images for: High frequency microcloning of Aloe vera and their true-to-type conformity by molecular cytogenetic assessment of two years old field growing regenerated plants
Source: Bot Stud. 2013 Oct 18;54:46. doi: 10.1186/1999-3110-54-46 (PMC5430365; doi:10.1186/1999-3110-54-46)

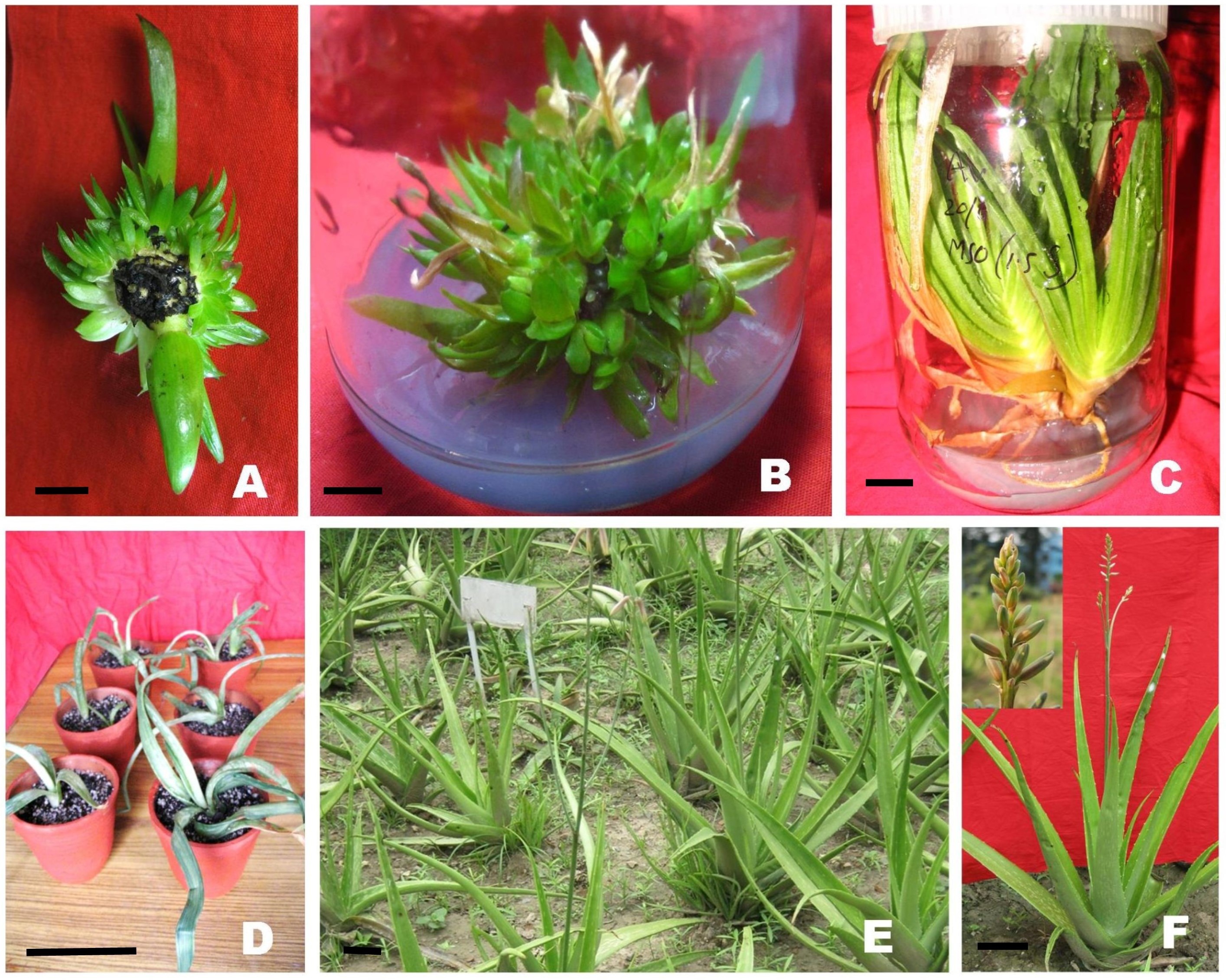

Supplement: Supplementary file 1 — Authors’ original file for figure 1 [file 40529_2013_96_MOESM1_ESM.jpeg]

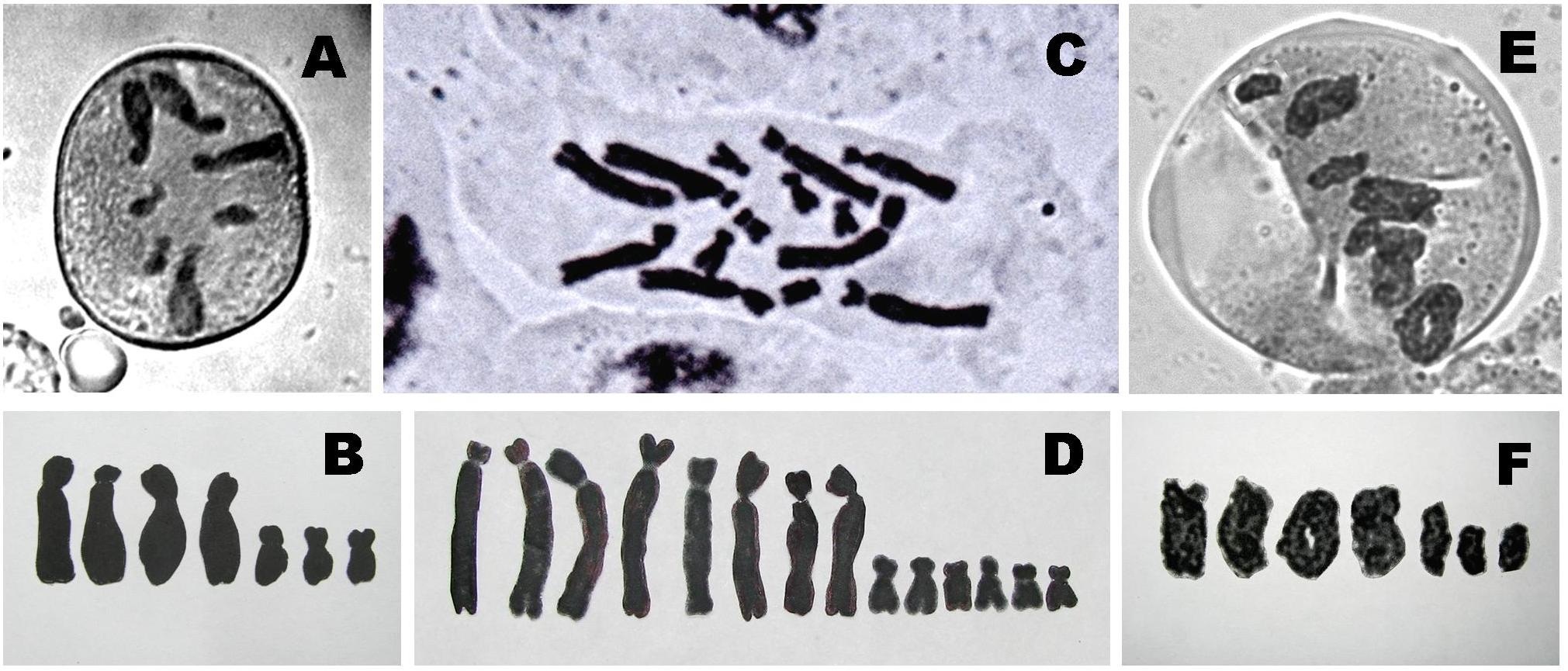

Supplement: Supplementary file 2 — Authors’ original file for figure 2 [file 40529_2013_96_MOESM2_ESM.jpeg]

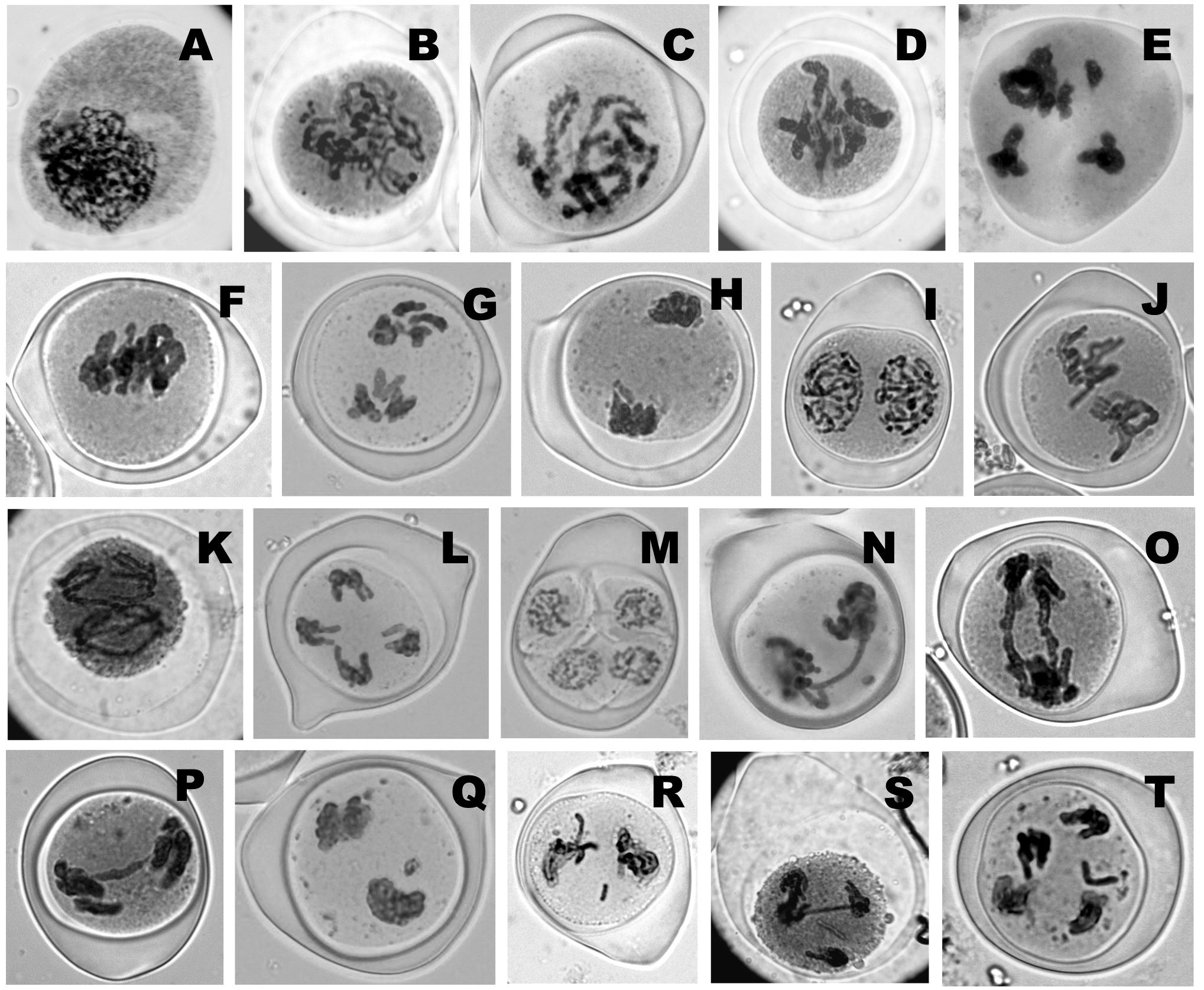

Supplement: Supplementary file 3 — Authors’ original file for figure 3 [file 40529_2013_96_MOESM3_ESM.jpeg]

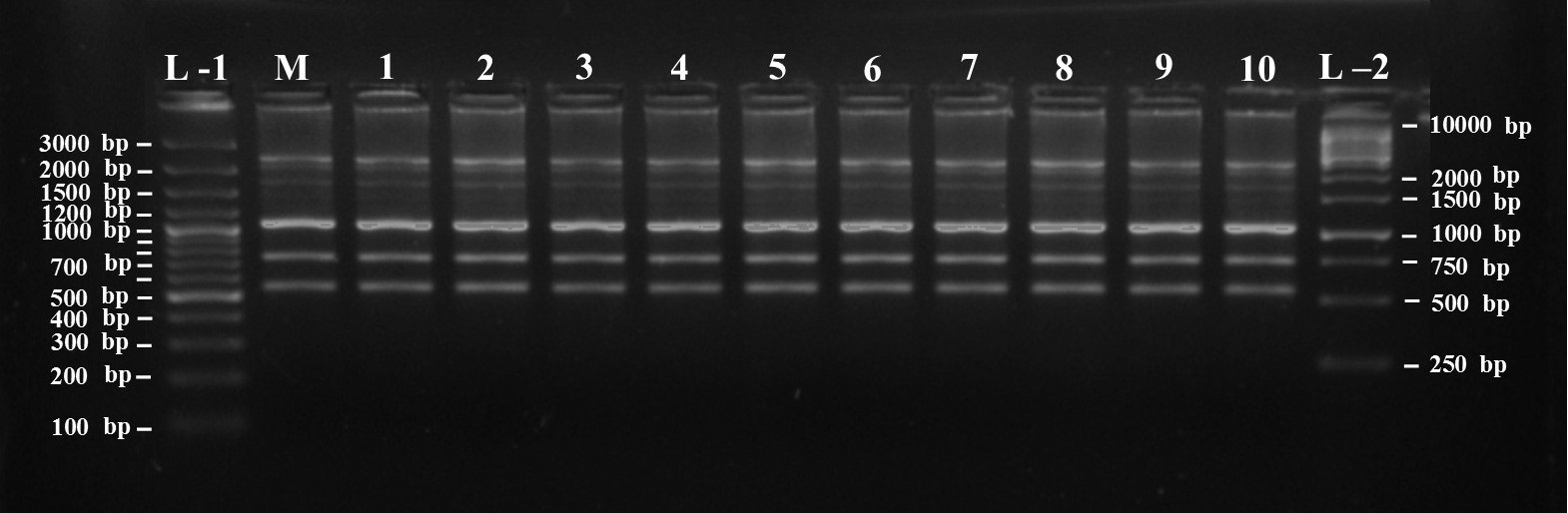

Supplement: Supplementary file 4 — Authors’ original file for figure 4 [file 40529_2013_96_MOESM4_ESM.jpeg]
